# Influence of Conventional and Ultrasonic-Assisted Extraction on Phenolic Contents, Betacyanin Contents, and Antioxidant Capacity of Red Dragon Fruit (*Hylocereus polyrhizus*)

**DOI:** 10.1155/2014/964731

**Published:** 2014-10-14

**Authors:** Nurul Shazini Ramli, Patimah Ismail, Asmah Rahmat

**Affiliations:** ^1^Department of Nutrition and Health Sciences, Faculty of Medicine and Health Sciences, Universiti Putra Malaysia, Serdang, 43400 Selangor, Malaysia; ^2^Department of Food Science, Faculty of Food Science and Technology, Universiti Putra Malaysia, Serdang, 43400 Selangor, Malaysia; ^3^Department of Biomedical Sciences, Faculty of Medicine and Health Sciences, Universiti Putra Malaysia, Serdang, 43400 Selangor, Malaysia

## Abstract

The aim of this study was to examine the effects of extraction methods on antioxidant capacities of red dragon fruit peel and flesh. Antioxidant capacities were measured using ethylenebenzothiozoline-6-sulfonic acid (ABTS) radical cation assay and ferric reducing antioxidant power assay (FRAP). Total phenolic content (TPC) was determined using Folin-Ciocalteu reagent while quantitative determination of total flavonoid content (TFC) was conducted using aluminium trichloride colorimetric method. Betacyanin content (BC) was measured by spectrophotometer. Red dragon fruit was extracted using conventional (CV) and ultrasonic-assisted extraction (UE) technique to determine the most efficient way of extracting its antioxidant components. Results indicated that UE increased TFC, reduced the extraction yield, BC, and TPC, but exhibited the strongest scavenging activity for the peel of red dragon fruit. In contrast, UE reduced BC, TFC, and scavenging activity but increased the yield for the flesh. Nonetheless, UE slightly increases TPC in flesh. Scavenging activity and reducing power were highly correlated with phenolic and flavonoid compounds. Conversely, the scavenging activity and reducing power were weakly correlated with betacyanin content. This work gives scientific evidences for the consideration of the type of extraction techniques for the peel and flesh of red dragon fruit in applied research and food industry.

## 1. Introduction

For decades, evaluation of antioxidant properties of plants food has been part of the basis for screening their potential in disease prevention and treatment [1]. Many studies on antioxidant capacities have been conducted and published, contributing to the body of knowledge. Even though* in vitro* studies do not guarantee the effectiveness* in vivo* and in human studies [2], it does give ideas and support to possible mechanism of action of the plant food in the biological system [3].

In order to maintain basic cell functions, free radicals are produced continuously in our body as a byproduct of energy metabolism and these free radicals are normally neutralized by the endogenous antioxidants. However, environmental exposure to poisons, smoke, and ionizing radiation can upset the balance of antioxidant system in the body, hence resulting in oxidative stress [4]. In retrospect, oxidative stress has been implicated in aging and the development of chronic diseases like cardiovascular diseases, diabetes, cancer, atherosclerosis, and Alzheimer's disease [5, 6].

Sample preparation and extraction techniques are very crucial in antioxidant studies since it can influence the recovery of antioxidant compounds from the plant materials and physiological function of the extracted substances. Many extraction techniques are available and have been used extensively by researchers in determination of antioxidant components and its capacity in plant food. Nowadays, novel extraction techniques have been developed to improve the extraction efficiency including microwave digestion, enzymatic extraction, and ultrasound-assisted extraction (UE) [7–9]. However, the applications of new extraction techniques on specific food need solid scientific evidence based on analysis of bioactive compounds and functional properties of the specific resulting food. The comparison between conventional and ultrasonic-assisted extraction is made since conventional method is commonly used in research while ultrasound assisted extraction is cost effective to be used commercially.

Red dragon fruit (*Hylocereus polyrhizus*) or sometimes called red pitaya has been comprehensively researched for its antioxidant potential especially the polyphenols and betacyanin. Betacyanin is a pigment that is responsible for the red purple color of the fruit [10]. A number of extraction techniques have been used for extracting bioactive compounds from dragon fruit like enzyme-assisted extraction using PectinexÂ Ultra SP-L, microwave, and ultrasonic extraction method [11–13]. However, to the best of our knowledge the comparison between conventional and ultrasonic-assisted extraction has not yet been done. Conventional extraction method using orbital shaker at high temperature involves longer extraction hours and hence resulted in degradation of heat-sensitive bioactive compounds. On the other hand, ultrasonic-assisted extraction is very simple and inexpensive [14, 15] and permits higher surface area contact between sample and solvent [16]. Thus, the present study is conducted to investigate the influence of conventional and ultrasound-assisted extraction using sonicator on antioxidant capacities of peel and flesh of red dragon fruit.

## 2. Materials and Methods

### 2.1. Materials

All chemicals and reagents used for spectrophotometric analysis were of analytical grade; absolute ethanol, Folin-Ciocalteu reagent and sodium carbonate, 2,2′-azino-bis(3-ethylbenzothiazoline-6-sulphonic acid) (ABTS), and potassium persulphate were purchased from Merck (Darmstadt, Germany); aluminium chloride hexahydrate (AlCl_3_ *·*6H_2_0) was from Fischer Scientific (NH, USA); gallic acid, acetate buffer, 2,4,6-tripyridyl-s-triazine (TPTZ), iron(III) chloride hexahydrate (FeCl_3_ *·*6H_2_0), and rutin were from Sigma-Aldrich (MO, USA).

### 2.2. Plant Material

Dragon fruits were obtained from four-year-old plants from Multi Rich farm in Mantin, Negeri Sembilan, Malaysia. The identification of the fruit was done by a botanist from Biodiversity Unit, Institute of Biosciences, Universiti Putra Malaysia. The voucher number is SK-2440/14 as previously described in Ramli et al. [17]. Dragon fruits were freshly harvested when reaching full ripening stage, 35–38 days after pollination, and immediately transported to the Nutritional Laboratory, Faculty of Medicine and Health sciences, Universiti Putra Malaysia.

### 2.3. Sample Preparation

The fruits were cleaned, and the residual water was removed using paper towel before being weighed. The flesh and peel were cut into small cubes and stored in −80°C freezer (Sony Ultra Low −80°C). The samples were spray-dried in a spray dryer (Niro, A/S, GEA, Germany) to reduce the moisture content for a more efficient extraction process. Sample preparation was conducted in reduced light condition in order to minimize the pigment loss. The spray-dried sample was then kept in airtight container before further analysis. The samples were extracted using either conventional method or ultrasonic-assisted extraction in order to compare which extraction method is more efficient in extracting the antioxidant compounds of the samples.

#### 2.3.1. Conventional Extraction (CV)

One g of the flesh was homogenized with 25 mL of distilled water for a few minutes at 1/25 (w/v). The mixture was shaken using shaking incubator at 200 rpm for 120 minutes at 50°C. Mucilaginous material was separated from the extract on a Büchner funnel through Whatman No. 1 filter paper to give a colored solution. The residue was reextracted with water for full pigment recovery. The mixture was then centrifuged at 6000 rpm for 15 minutes at room temperature and supernatant was saved. For the peel, 55 mL of distilled water was added to 1 g of the sample at 1/55 (w/v). The extraction for both flesh and peel was conducted in triplicate (*n* = 3). Then, the supernatants were used for the determination of total phenolic and total flavonoid content, betacyanin content, and antioxidant activity using ferric reducing antioxidant power (FRAP) and ABTS^+^ radical scavenging assay.

#### 2.3.2. Ultrasonic-Assisted Extraction (UE)

One g of the pulp was homogenized with 25 mL of distilled water for a few minutes at 1/25 (w/v). The mixture was then placed in ultrasonic bath and sonicated at 50 kHz for 30 minutes at ambient temperature (25°C). Mucilaginous material was separated from the extract on a Büchner funnel through Whatman No. 1 filter paper to give a colored solution. The residue was reextracted with water for full pigment recovery. The mixture was then centrifuged (Universal 32r, Hettich Zentrifugen, England) at 6000 rpm for 15 minutes at room temperature and supernatant was saved. For the peel, 55 mL of distilled water was added to 1 g of the sample at 1/55 (w/v). The extraction for both flesh and peel was conducted in triplicate. Then, the supernatants were used for determination of total phenolic and total flavonoid content, betacyanin content and determination of antioxidant activity using ferric reducing antioxidant power (FRAP) and ABTS^+^ radical scavenging assay.

### 2.4. Extraction Yield

Extraction yield was measured based on a method from Zhang et al. [18]. The resulting extract was lyophilized in a freeze dryer (The Virtis Company, Inc., Gardiner, New York). Then the freeze dried extract was weighed. Extraction yield was calculated per 1 g of raw material.

### 2.5. Betacyanin Content

Betacyanin content was quantified according to the method by Wybraniec and Mizrahi [19] with slight modifications whereby the mean molar absorptivity was set at 65000 for betanin instead of 60000. The betacyanin content of dry extracts was determined by the spectrophotometric method and expressed as betanin equivalents (mg/100 g of dry extracts) based on the formula below:

(1) Concentration  of  betacyanins  (mg/100 g  dry  extracts)  =A538(MW)V(DF)×100εLW,

where *A*_538_ = absorbance  at  538 nm, *L* = path  length, 1.0 cm, DF = dilution  factor,*V* = pigment  solution  volume  (mL), and *W* = dried  pigment  weight  (g). For betanin, *ε* (molar extinction coefficient) = 65,000 and molecular weight (MW) = 550.

### 2.6. Determination of Total Phenolic Content

Determination of total phenolic content was carried out using the Folin-Ciocalteu's reagent as described by Singleton and Rossi [20] with slight modifications whereby the steps of heating at 100°C in a water bath are removed and the incubation time is increased from 1 minute to 90 minutes. The red dragon fruit extract (200 *μ*L) was mixed with 1.5 mL of Folin-Ciocalteu reagent (previously diluted tenfold with distilled water) and allowed to stand at room temperature for 5 min. A 1.5 mL of sodium carbonate solution (60 g/L) was added to the mixture. The tubes were vortexed, covered with parafilm, and allowed to stand for 90 minutes. The absorbance was measured at 750 nm using Secomam's RS232 ultraviolet-visible (UV-vis) spectrophotometer (Cedex, France) after a 90 min incubation at room temperature. The total phenolic content was calculated using regression equations from standard curve of gallic acid (20–200 *μ*g/mL in water). The results were expressed as mg% dry basis.

### 2.7. Determination of Total Flavonoid Content

The flavonoid content was estimated by using aluminium trichloride colorimetric with slight modification based on the method by Quettier-Deleu et al. [21] whereby 5 mL of 2% AlCl_3_, 6H_2_O has been reduced to 1 mL. In brief, 1 mL of extract solution was added to 1 mL of 2% AlCl_3_, 6H_2_O. The absorbance was measured after 10 min of incubation at 430 nm. The result was expressed in mg% dry basis by comparison with standard rutin treated in the same condition.

### 2.8. Determination of Antioxidant Capacity

#### 2.8.1. ABTS^+^ Scavenging Assay

The ABTS^+^ radical scavenging assay was done according to the method by Hurtado et al. [22]. The mixture of 7 mM ABTS and 2.45 mM potassium persulphate was kept in an amber bottle and incubated in the dark at room temperature for 16 hours to produce ABTS^+^ radical cation. The solution was then diluted with distilled water to get absorbance of 0.70 ± 0.1 at 734 nm, measured using UV-Vis spectrophotometer (SECOMAM, France). Then, 20 *μ*L of sample extract or standard (Trolox) to 1.0 mL ABTS^+^ solution was added and mixed thoroughly. The sample was diluted with the respective extraction media to give 20–80% inhibition of the blank. Trolox standard at a final concentration of 0.004–0.24 mM was prepared in distilled water and assayed under the same conditions. The Trolox equivalent antioxidant capacity (TEAC) of the red dragon fruit extracts was calculated based on the inhibition exerted by the standard Trolox solution at 6 min. Consider the following:

(2) $$\begin{array}{c} \%\;\text{of}\;\text{scavenging}\;\text{capacity}=1-\left(\frac{{\text{Absorbance}}_{\text{sample}}}{{\text{Absorbance}}_{\text{control}}}\right)\times 100. \end{array}$$

#### 2.8.2. Ferric-Reducing Antioxidant Power Assay

Determination of Ferric-reducing power (FRAP) was carried out using a method described by Benzie and Strain [23] with some modifications whereby the incubation time was changed from 15 seconds to 15 minutes and samples were diluted before it was mixed with FRAP reagent. The FRAP reagent was prepared by mixing 0.3 M acetate buffer (pH 3.6), 0.01 M of TPTZ, and 0.02 M of FeCl_3_ *·*6H_2_O with a ratio of 10 : 1 : 1 at 37°C. Red dragon fruit extract (0.2 mL) was mixed with 3 mL of FRAP reagent and the mixture was incubated at 37°C. Triplicate measurements were conducted and the changes in absorbance were measured at 593 nm after initial time and at 15 min. Trolox concentrations of 50–1000 *μ*M were used for calibration of the FRAP assay, and antioxidant power was expressed as mM of TEAC per 100 g dry weight.

### 2.9. Statistical Analysis

All data were analyzed using SPSS for Windows version 19.0. Independent *t*-test is used to test whether there are significant differences in total phenolic, total flavonoid, betacyanin content, and antioxidant capacities between the two extraction methods of red dragon fruit peel and flesh. Pearson correlation coefficient was used to test the correlation between antioxidant capacities and total phenolic, total flavonoid, and betacyanin content. All data are expressed as means ± standard error of mean. Statistical significance was set at *P* = 0.05.

## 3. Results and Discussion

### 3.1. Extraction Yield

Table 1 summarizes the extraction yield, betacyanin content, and antioxidant capacities of red dragon fruit peel and flesh. The highest extraction yield was obtained from the peel of red dragon fruit extracted using conventional extraction method (95.25%) whereas the flesh extracted using ultrasonic-assisted extraction had the highest yield (90.08%). The peels of studied sample contained higher concentration of pectin [24] and may require longer extraction time to complete the separation of compound, for the compound to be fully diffused into the solvent, as applied in CV for 2 hours compared to UE for only 30 minutes. The flesh of red dragon fruit had the highest extraction yield using UE. It was found that ultrasound-assisted extraction of polyphenols from orange was able to reduce energy input from the higher extraction yield in shorter time [16]. UE produces ultrasonic waves that attack the integrity of plant cellular walls. This resulted in increased permeability of cytoplasmic membranes as evidenced by scanning electron microscopy [25] and more solvent can enter into the plant cell while causing the release of more compounds into the solvent [26].

### 3.2. Betacyanin Content

Results in Table 1 showed CV was more effective for extracting betacyanin from the peel and flesh. Betacyanin is very sensitive to high temperature. It was found that almost 90% of the pigment retention was reduced with the increasing of temperature from 25°C to 75°C [27]. Surprisingly, the CV did not contribute to the decrease in total betacyanin content in this present study. In fact, betacyanin content detected in this study was significantly higher compared to study by Tang and Norziah [27] whereby they conducted the extraction in room temperature (25°C) and found 10.1 ± 0.6 mg/100 g dry weight and 6.7 ± 0.2 mg/100 g dry weight in flesh and peel, respectively.

### 3.3. Total Phenolic Content

**Figure 1:**
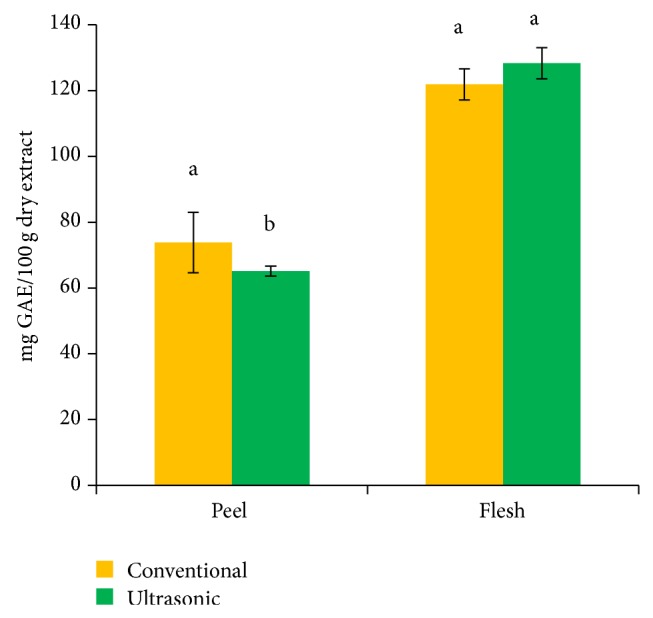
Total phenolic contents extracted from red dragon fruit of different parts by using different methods. (Mean with different alphabets for the same plant parts are significantly different (*P* < 0.05).)

Figure 1 shows mean TPC of spray-dried peel and flesh of red dragon fruit extracted using conventional and ultrasonic-assisted extraction methods. The results from this study found that TPC was significantly higher in peel extracted using CV (73.84 ± 15.94 mg% dry basis) compared to peel extracted by UE (65.16 ± 1.52 mg% dry basis). On the other hand, no difference between the two extraction methods for the flesh (121.86 ± 4.89 mg% dry basis and 128.30 ± 8.17 mg% dry basis extracted in CV and UE resp.). It is possible that the use of CV increased the release of bound phenolic compound from the peel compared to UE method. Some authors [28, 29] have postulated that the loss of TPC in dried samples at high temperature could be resulted from degradation of phenolic compounds due to thermal effect and/or reduced free phenolic compound. Besides, the reduction of TPC also contributed to the loss betacyanin as betacyanin also contained phenol structure in molecule. However, the present study found higher total phenolic compounds which may be resulted from increased betacyanin content when extracted using CV. Previous research reported that 70% ethanolic extract of red dragon fruit contained 28.16 and 19.72 mg% dry basis in peel and pulp, respectively [30]. Since the present study found more than 100 times of TPC in water extract, it seems that most of phenolic compounds in red dragon fruit are water soluble.

### 3.4. Total Flavonoid Content

**Figure 2:**
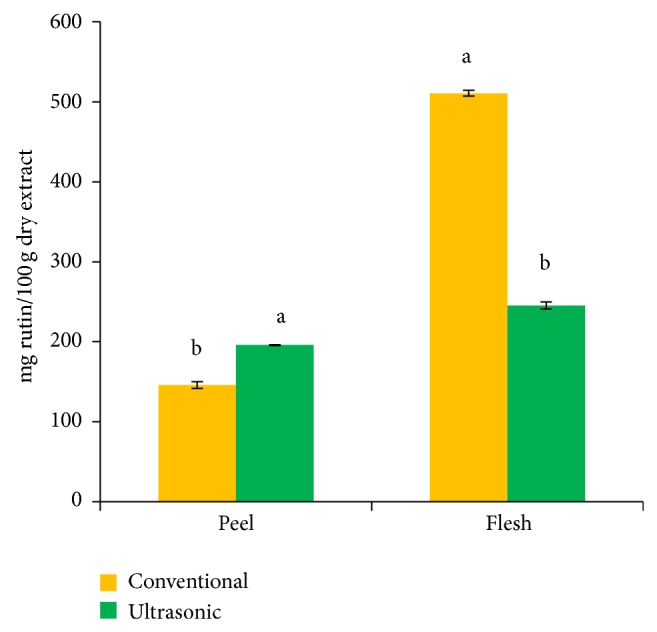
Total flavonoid contents extracted from red dragon fruit of different parts by using different methods. (Mean with different alphabets for the same plant parts are significantly different (*P* < 0.05).)

Figure 2 illustrates mean TFC of red dragon fruit peel and flesh extracted using CV and UE. TFC in peel was higher when extracted using UE compared to CV (195.82 ± 55.0 mg% dry basis and 145.88 ± 2.33 mg% dry basis extracted in CV and UE, resp.). Contrarily, flesh extracted by using CV had higher TFC (510.69 ± 7.54 mg% dry basis compared to UE (245.31 ± 6.04 mg% dry basis). Flavonoid is a subset of phenolic compounds [31]; however, high flavonoid content does not lead to high total phenolic content in this present study.

### 3.5. Antioxidant Capacity Assay

#### 3.5.1. FRAP Assay

Briefly, the antioxidant power is equal to reducing capacity. It can be seen from the results in Table 1 that flesh exhibited FRAP values of 620 ± 54.08 *μ*mol Fe2+/g dry extract using UE and 609.17 ± 54.51 *μ*mol Fe2+/g dry extract using CV. It was noted that peel showed the lowest FRAP values of 200.83 ± 66.35 *μ*mol Fe2+/g dry extract using CV and 255 ± 86.49 *μ*mol Fe2+/g dry extracted using UE. However, the present study revealed no significant difference between the two extraction methods (*P* > 0.05) for both peel and flesh in contrast with reports from a previous study [16]. Besides, Rebecca et al. [32] reported that the reducing power of red dragon fruit increased when the concentration of the sample was increased from 0.03125 g, 0.0625 g, 0.125 g, 0.25 g, and 0.5 g.

#### 3.5.2. ABTS^+^ Scavenging Assay

Determination of ABTS^+^ scavenging assay was carried out based on the decolorization of the preformed radical monocation of 2,2′-azinobis-(3-ethylbenzothiazoline-6-sulfonic acid) (ABTS) produced through the reaction between ABTS and potassium persulfate. This method has been reported to be relevant for both lipophilic and hydrophilic antioxidants [31]. As shown in Table 1, the scavenging effect of red dragon fruit peel ranged from 3.04 ± 0.36% to 6.06 ± 0.03% for spray dried sample extracted by CV and UE, respectively. A significant difference was found between the two extraction methods (*P* < 0.05). Similarly, the scavenging effect for flesh shows significant difference between CV and UE. For flesh, the scavenging effect was 5.73 ± 0.33% and 0.85 ± 0.55% when extracted using CV and UE, respectively. In comparison with previous study, the antioxidant assay measured by ABTS for red dragon fruit flesh and peel was found to be 1,029 and 815.03 *μ*g Trolox equivalents/g FM [11]. The scavenging effect of dragon fruit peel extracted using UE (6.06 ± 0.03%) and dragon fruit flesh extracted using CV (5.73 ± 0.33%) displayed higher scavenging activity compared to Sapodilla fruit (4.30% and 5.36% for the flesh and peel, resp.) [33]. Findings from previous study pointed out the suitability of ultrasonic-assisted extraction in the preparation of antioxidant-rich plant extracts especially in orange peel [16]. While UE conducted at ambient temperature for 30 minutes resulted in about 50% increase in the scavenging activity of the peel, the CV of the flesh at 50°C for 2 hours resulted in four times increase in the scavenging activity. Interestingly, the effects of time and temperature during sample preparation and extraction on antioxidant capacities are unique to the specific plant parts.

### 3.6. Correlation between Phenolic/Flavonoid Compound and Antioxidant Activity

The role of phenolic compounds as antioxidants has been recognized by means of their ability to donate electron and formed a stable radical intermediates. Esquivel et al. [34] reported that betacyanin was the major compound that contributed to the antioxidant activity of red dragon fruit. The present study, however, found that scavenging activity and reducing power were weakly correlated with betacyanin content (*r* ^2^ = 0.382 and *r* ^2^ = 0.381, resp.) (Table 2). Furthermore, the scavenging activity and reducing power were highly correlated with phenolic (*r* ^2^ = 0.810 and *r* ^2^ = 0.990, resp.) and flavonoid compounds (*r* ^2^ = 0.549 and *r* ^2^ = 0.890, resp.) (Table 2). This finding was in agreement with previous study whereby total phenolic and total flavonoid content of papaya extract were strongly correlated with antioxidant activity [30]. It does show that flavonoid was an important factor contributing to the radical scavenging activity in this present study.

## 4. Conclusions

The findings of this study suggest that ultrasonic-assisted extraction significantly reduces the extraction yield but increases the flavonoid content and scavenging activity for the peel of red dragon fruit. Conversely, ultrasonic-assisted extraction increases the extraction yield of the flesh but reduces total flavonoid content and scavenging activity. Hence, it can be seen that the influence of extraction method depends upon the physical property of the sample. This work gives scientific evidences for the consideration of the use of UE for the peel of red dragon fruit in applied research and food industry. Even though the extraction yield is an important factor to consider for production of functional food in large scale manufacturing, it is more crucial to take into consideration its biological effects. Therefore, the application of extraction methods for food or dietary supplement warrant further research before being produced in large scale in order to identify the most efficient extraction techniques for extracting its antioxidants.

## Figures and Tables

**Table 1:** Extraction yield, betacyanin content, scavenging activity, and reducing power of peel and flesh from red pitaya of different extraction methods.

Samples	Peel			
	Extraction yield	Betacyanin content	Scavenging activity	FRAP
	(%)	(mg/100 g dry extracts)	(%)	(*μ*mol Fe^2+^/g dry extract)
UE	47.07^a^	17.64 ± 0.03^b^	6.06 ± 0.03^a^	255 ± 49.94^a^
CV	95.25^b^	18.67 ± 0.50^a^	3.04 ± 0.36^b^	200.83 ± 38.3^a^

	Flesh			

UE	90.08^a^	71.34 ± 1.00^b^	0.85 ± 0.55^b^	620 ± 54.08^a^
CV	73.27^b^	82.79 ± 0.55^a^	5.73 ± 0.33^a^	609.17 ± 54.51^a^

UE: ultrasonic-assisted extraction.

CV: conventional extraction.

Values are expressed as mean ± SEM (*n* = 3).

Means within a column followed by different letters within each fruit part were significantly different at *P* < 0.05.

**Table 2:** Correlation of the phytochemical contents with different antioxidant indicators.

	Scavenging activity	Reducing power
Total phenolic content	0.810	0.990
Total flavonoid content	0.549	0.890
Betacyanin content	0.382	0.381

Correlation is significant at the 0.05 level (1-tailed).
